# Serum Vitamin D Levels in Pregnant Women: The Role of Supplementation on Preterm Infant Outcomes

**DOI:** 10.1155/jp/8348907

**Published:** 2026-08-28

**Authors:** Maryam Zakerihamidi, Forough Rakhshanizadeh, Fahimeh Afzaljavan, Hassan Boskabadi

**Affiliations:** ^1^ Department of Midwifery, To.C., Islamic Azad University, Tonekabon, Iran, azad.ac.ir; ^2^ Department of Pediatrics, Faculty of Medicine, Mashhad University of Medical Sciences, Mashhad, Iran, mums.ac.ir; ^3^ Department of Obstetrics and Gynecology, School of Medicine, Mashhad University of Medical Sciences, Mashhad, Iran, mums.ac.ir

**Keywords:** mothers, neonatal resuscitation, preterm infants, respiratory distress syndrome, supplement, vitamin D

## Abstract

**Objective:**

Vitamin D plays a crucial role in maternal and infant health. This study was aimed at evaluating and comparing serum vitamin D levels and clinical outcomes between preterm mothers who received vitamin D supplements and those who did not.

**Methods:**

In a cross‐sectional study, a total of 328 women were enrolled, of whom 310 mother–infant pairs were included in the final analysis. The supplementation group consisted of mothers who consumed at least 1000 international units of vitamin D daily for a minimum of 1 month prior to delivery. Clinical characteristics and neonatal outcomes were compared between the supplementation and nonsupplementation cohorts.

**Results:**

The mean serum vitamin D levels were significantly higher in the supplementation group for both mothers (23.88 ± 15.66 vs. 12.68 ± 7.37 ng/mL) and their infants (17.55 ± 12.23 vs. 10.38 ± 7.35 ng/mL; p <0.001 for both). Infants in the nonsupplementation group required resuscitation in the delivery room significantly more often (p = 0.019). Furthermore, the incidence of respiratory distress syndrome (p = 0.002) and respiratory problems (p = 0.004) were significantly higher in the nonsupplemented group. No statistically significant differences were observed regarding one‐ and fifth‐minute Apgar scores (p = 0.200 and p = 0.117), birth weight (p = 0.343), or neonatal mortality (p = 0.062).

**Conclusions:**

Maternal vitamin D supplementation is associated with higher serum vitamin D levels in both mothers and preterm infants, as well as a reduced prevalence of adverse neonatal respiratory outcomes. These findings underscore the clinical importance of prenatal vitamin D supplementation to promote maternal and neonatal health, particularly in populations with high rates of vitamin D deficiency.

**Summary**

• What is known?
  ◦ A strong correlation between maternal and infant serum vitamin D levels.
  ◦ Preterm infants may require more vitamin D in the first month postdelivery compared to term infants, as they are at a heightened risk for vitamin D deficiency due to limited placental transfer during the last trimester of pregnancy.
• What is new?
  ◦ Maternal vitamin D supplementation is associated with significantly higher serum vitamin D levels in mothers and their preterm infants and a lower risk of adverse neonatal outcomes. Vitamin D supplementation during pregnancy may be associated with better maternal and neonatal health in preterm populations. With the use of vitamin D supplementation in the mother, the proportion of mothers/infants with sufficient vitamin D levels was higher (34% vs. 8.8%), and the normalization of vitamin D in the infant was six times higher (19% vs. 3.5%). In addition, the need for resuscitation in neonates was lower in the case group.

## 1. Introduction

Vitamin D plays a fundamental role in growth and development, maintaining the health of the bones of both mother and fetus [1] and regulating calcium homeostasis and blood levels. Vitamin D levels during pregnancy can significantly impact the mother′s and the fetus′s health. Numerous studies have highlighted the association of 25(OH)D levels with gestational complications, including gestational hypertension, preeclampsia [2], gestational diabetes [3], and preterm labor. Additionally, low vitamin D levels have been linked to fetal complications such as intrauterine growth restriction [2], allergies, obesity, and cardiac dysfunction [4]. The prevalence of vitamin D deficiency remains alarmingly high, affecting approximately 30%–93% of pregnant women in countries such as China, Turkey, India, Saudi Arabia, and Iran. In Iran specifically, reports indicate a deficiency rate of 40%–80% [1]. Notably, about 75% of women experiencing preterm labor exhibit significant vitamin D deficiency [5].

While most studies report a positive correlation between maternal and neonatal serum vitamin D levels [6], some have found weaker associations in certain populations, possibly due to confounding factors like diet or sunlight exposure [7]. The fetus is entirely dependent on the mother′s 25(OH)D level for bone growth and embryonic–placental integration, as this nutrient influences placental mineral transport and hormonal balance [8].

Thus, it is essential to address maternal vitamin D deficiency, as it is the primary contributor to neonatal vitamin D deficiency [9]. Recent research indicates that vitamin D deficiency in premature infants is linked to various adverse outcomes [5]. Correcting maternal vitamin D levels may reduce the incidence of prematurity and mitigate these infantile complications [10]. Vitamin D supplementation is recommended as part of routine prenatal care to improve outcomes for mothers and neonates. Moreover, evidence suggests that vitamin D supplementation can reduce the risk of hypertension during pregnancy and enhance neonatal metrics such as height and head circumference at birth [11]. Levels of 25(OH)D increase during pregnancy, doubling serum concentrations in the third trimester compared to nonpregnant women or those recently giving birth [12]. Preterm infants may require more vitamin D in the first month postdelivery compared to term infants, as they are at a heightened risk for vitamin D deficiency due to limited placental transfer during the last trimester of pregnancy [6]. Studies have shown that most preterm infants exhibit severe vitamin D deficiency, which is particularly pronounced in cases of neonatal death compared to surviving babies [13]. Therefore, measuring vitamin D levels in the umbilical cord blood could aid in predicting the prognosis of premature infants.

Some studies have demonstrated the protective effects of adequate maternal vitamin D levels against preterm labor [14] and suggested that daily vitamin D supplementation at doses exceeding 10 *μ*g/day is necessary to enhance maternal and neonatal health outcomes [15]. Given the high prevalence of deficiency in preterm populations and the established link between vitamin D status and neonatal prognosis, this study was aimed at evaluating and comparing serum vitamin D levels and neonatal outcomes in preterm mothers with a gestational age less than 37 weeks based on their supplementation status.

## 2. Methods

A cross‐sectional study was conducted from 2019 to 2024 at Ghaem Educational Hospital, affiliated with the Mashhad University of Medical Sciences. A convenience sampling method was employed to assess serum vitamin D levels in 328 mothers exhibiting signs of preterm labor, both with and without vitamin D supplementation, and their preterm infant outcomes. The sample size was calculated a priori based on similar studies to detect differences in binary outcomes, such as respiratory distress syndrome (RDS), with expected rates of ~60% in the nonsupplemented group and ~43% in the supplemented group (difference ~17%). Using the formula for two independent proportions with *α* = 0.05 and power = 0.85, the minimum sample size was ~135 per group. Accounting for 20% attrition, we aimed for at least 324 participants [16].

This study investigated pregnant women presenting signs of labor before 37 weeks of gestation who were referred to Ghaem Educational Hospital of Mashhad University of Medical Sciences during the years 2019–2024. Informed consent was obtained from all participants. Exclusion criteria included mothers taking anticonvulsant medications, infants with congenital anomalies or infections, and cases with insufficient maternal or neonatal blood samples for laboratory testing.

After obtaining approval from the ethics committee and securing informed consent from pregnant mothers, we assessed vitamin D supplementation in mothers presenting labor pains and a gestational age of less than 37 weeks. Supplementation was self‐reported via questionnaire at admission.

The study population included 328 mothers. Participants were classified into two groups based on vitamin D supplementation: (1) those who received at least 1000 IU of vitamin D daily for a minimum duration of 1 month and (2) those who did not take supplements or took them for less than 1 month. The minimum exposure threshold for inclusion in the supplementation group was defined as receiving ≥ 1000 IU of vitamin D daily for at least 1 month.

At delivery, serum vitamin D levels were measured in mothers and their infants′ umbilical cord blood. The prepared samples were centrifuged, and the serum was stored at −20°C before laboratory evaluation. Vitamin D levels were quantified using an ELISA reader (RT2100c, Germany) and an ELISA washing device.

Vitamin D levels were categorized based on Endocrine Society guidelines [17]: Levels less than 30 ng/mL were classified as vitamin D deficiency, while levels equal to or greater than 30 ng/mL were considered sufficient. Additionally, we defined severe vitamin D deficiency as less than 10 ng/mL, moderate deficiency as 10–19.99 ng/mL, and mild deficiency as 20–29.99 ng/mL.

Comprehensive data on newborn characteristics, including birth weight, age, sex, gestational age, Apgar score, and need for resuscitation at birth, were collected alongside maternal history (age and delivery method) and laboratory results. This information was systematically recorded using a checklist, and the characteristics of the mothers and infants in the two groups were subsequently compared.

Neonatal outcome included infant characteristics at birth, respiratory problems, RDS, and death. Respiratory problems in infants were diagnosed based on clinical signs observed within the first day of birth, including tachypnea, use of accessory muscles for breathing, grunting, and nasal flaring. Neonatal RDS was diagnosed based on at least two of the following criteria: (A) signs of respiratory distress (e.g., nasal flaring, grunting, tachypnea, and retractions) after delivery, requiring respiratory support (e.g., nasal CPAP or NIPPV with PEEP > 6 cm H_2_O and/or FiO_2_ > 30%) for more than 24 h; (B) radiographic evidence of RDS; and (C) requirement for surfactant replacement therapy.

## 3. Statistical Analysis

Data were analyzed using SPSS Version 23 (IBM Corp., Chicago, IL, United States). Descriptive statistics, including number and percent or mean and standard deviation, were used to report the study population characteristics. The distribution of the variables was investigated using the Kolmogorov–Smirnov test. The independent *t*‐test was performed to compare the means of quantitative variables between the groups, whereas the chi‐square test was used to compare qualitative variables. A binary logistic regression analysis was applied to estimate odds ratios (ORs) and 95% confidence intervals (CIs). Multiple logistic regression was used to explore factors influencing neonatal vitamin D deficiency, adjusting for main confounders such as gestational age and prenatal corticosteroid use by the mother. A *p* value of less than 0.05 was considered statistically significant.

## 4. Results

Of the 328 participants in the study, 18 mothers were excluded, including intake of anticonvulsant drugs (*n* = 3), mothers′ inadequate samples for testing (*n* = 6), fetal congenital anomalies (*n* = 3), infections (*n* = 3), and infants′ inadequate samples for testing (*n* = 3). It was performed on 310 mothers and infants, including 173 mothers who took vitamin D and 137 whose mothers did not.

Table 1 summarizes the study population baseline characteristics. Maternal age (*p* = 0.798) and parity number (*p* = 0.725) were similar between groups. The mean gestational age was 31.61 ± 2.53 and 31.69 ± 2.88 weeks in mothers with and without vitamin D supplementation, without a significant difference between groups (*p* = 0.999). The mean duration of 1000 units of vitamin D supplementation per day was 2.5 months. Serum vitamin D levels in mothers (*p* < 0.001) significantly differed between groups with and without supplementation. There were significant relationships between vitamin D consumption by the mother with maternal vitamin D levels (*p* < 0.001). The normal vitamin D levels were observed in 34.1% (*n* = 59) and 8.8% (*n* = 12) mothers with and without supplementation. Besides, severe deficiency was observed in 21.4% (*n* = 37) and 46% (*n* = 63) of mothers with and without supplementation. Serum vitamin D levels in infants were significantly different between groups with and without supplementation (*p* < 0.001, OR = 1.13, 95% CI [1.08–1.18]).

**Table 1:** Comparing the characteristics of the study population between groups with and without taking vitamin D supplementation.

Variables		Mothers taking vitamin D (*n* = 173)	Mothers without taking vitamin D (*n* = 137)	*p*value ^∗^
Maternal age (year)		29.51 ± 6.55	29.32 ± 6.24	0.798
Parity		2.18 ± 1.22	2.23 ± 1.25	0.725
Maternal vitamin D (ng/mL)		23.88 ± 15.66	12.68 ± 7.37	**< 0.001**
Prenatal corticosteroid use by the mother	Yes	94 (52.2)	86 (47.8)	0.232
	No	37 (61.7)	23 (38.3)	

Infant sex	Male	84 (45.9)	73 (53.3)	0.191
	Female	99 (54.1)	64 (46.7)	

Delivery method	Vaginal delivery	72 (41.6)	45 (33)	0.194
	Cesarean	101 (58.4)	92 (67)	

Maternal vitamin D status	Normal	59 (34.1)	12 (8.8)	**< 0.001**
	Mild	26 (15)	14 (10.2)	
	Moderate	51 (29.5)	48 (35)	
	Severe	37 (21.4)	63 (46)	

*Note:* Significant *p* values are shown in bold.

^∗^ *p* value of less than 0.05 was regarded as statistically significant.

Table 2 describes the short‐term outcome of the infant in two groups of mothers with and without vitamin D supplementation. Serum vitamin D levels in infants were significantly different between groups with and without supplementation (*p* < 0.006, OR = 1.18, 95% CI [1.05–1.32]). Similarly, a significant relationship was observed between vitamin D consumption and neonatal vitamin D deficiency (*p* < 0.001). Normal vitamin D levels were observed in 19% (*n* = 33) and 3.5% (*n* =  5) of infants, whereas 36.4% (*n* = 63) and 62.2% (*n* = 85) of infants had severe deficiency in groups with and without supplementation, respectively.

**Table 2:** Comparing infant outcomes between groups with and without supplementation.

Variables	Mothers taking vitamin D (*n* = 173)	Mothers without taking vitamin D (*n* = 137)	OR (95% CI)	*p*value ^∗^
Gestational age (week)	31.61 ± 2.53	31.69 ± 2.88	1.00 (0.921–1.081)	0.999
First‐minute Apgar score	6.76 ± 1.86	6.45 ± 2.36	1.071 (0.964–1.191)	0.200
Fifth‐minute Apgar score	8.15 ± 1.48	7.85 ± 1.94	1.111 (0.974–1.267)	0.117
Birth weight (grams)	1481.74 ± 505.46	1536.25 ± 511.80	1.000 (0.999–1.000)	0.343
Neonatal vitamin D (ng/mL)	17.55 ± 12.23	10.38 ± 7.35	1.181 (1.049–1.329)	**0.006**
Need for CPR in the delivery room				
No	137 (79.2)	95 (69.3)	Reference	**0.019**
Yes	36 (20.8)	42 (30.7)	1.87 (1.11–3.15)	
Neonatal death				
No	163 (94.2)	117 (87.3)	Reference	0.062
Yes	10 (5.8)	17 (12.7)	2.095 (0.965–4.548)	
Neonatal vitamin D deficiency				
Normal	33 (19)	5 (3.5)	Reference	
Mild	28 (16.2)	16 (11.7)	3.97 (0.001–13.33)	**0.026**
Moderate	49 (28.4)	31 (22.6)	4.59 (1.47–14.28)	**0.009**
Severe	63 (36.4)	85 (62.2)	10.31 (3.47–30.30)	**< 0.001**
Respiratory problem				
No	89 (50.9)	44 (35.2)	Reference	**0.004**
Yes	85 (49.1)	81 (64.8)	1.93 (1.20–3.08)	
Respiratory distress syndrome				
No	94 (57.0)	46 (39.3)	Reference	**0.002**
Yes	71 (43.0)	71 (60.7)	2.04 (1.20–3.31)	

*Note:* Significant *p* values are shown in bold.

^∗^ *p* value of less than 0.05 was regarded as statistically significant.

**Figure 1:**
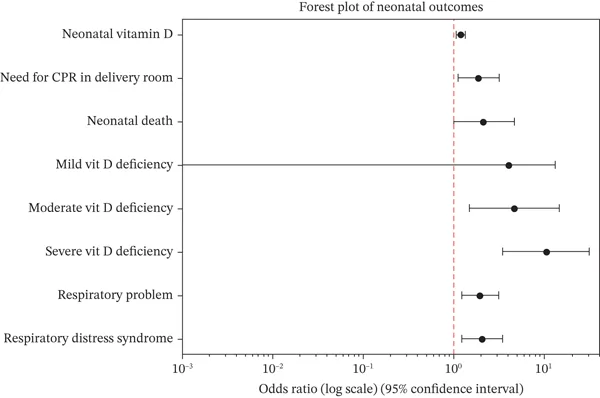
Forest plot of odds ratios and 95% confidence intervals for neonatal outcomes.

Although the need to resuscitate the infant in the delivery room was significantly higher in groups without vitamin D supplementation (*p* = 0.019, OR = 1.87, 95% CI [1.11–3.15]), first and fifth‐minute Apgar scores (*p* = 0.200 and *p* = 0.117), birth weight (*p* = 0.343), and infant death after delivery (*p* = 0.062) did not indicate a significant difference between groups. However, respiratory problems (*p* = 0.004, OR = 1.93, 95% CI [1.20–3.08]) and RDS (*p* = 0.002, OR = 2.04, 95% CI [1.20–3.31]) were significantly higher in groups without supplementation (Table 2 and Figure 1).

Multivariate logistic regression analysis (Table 3) demonstrated that maternal vitamin D supplementation was an independent protective factor against neonatal vitamin D deficiency (*p* = 0.001, OR = 6.19, 95% CI [2.08–18.43]), after adjusting for potential confounders, including gestational age and prenatal corticosteroid administration.

**Table 3:** Multiple logistic regression for factors influencing neonatal vitamin D deficiency.

Variable	Multivariate analysis	
	OR (95% CI)	*p*value
Group	6.192 (2.08–18.43)	0.001
Prenatal corticosteroid use by the mother	1.774 (0.799−4.040)	0.172
Gestational age	0.921 (0.769−1.102)	0.376

## 5. Discussion

Vitamin D deficiency is a subject of attention in pregnancy and neonatal outcomes due to its impact on maternal complications as well as neonatal growth and development. This study focused on preterm deliveries and compared neonatal outcomes between two groups of mothers with and without vitamin D supplementation. Our study found that vitamin D supplementation for at least 1 month (mean duration: 2.5 months) was associated with a lower risk of severe deficiency in infants (up to 10‐fold lower odds). Furthermore, this supplementation was associated with a substantial reduction in the risk of neonatal vitamin D deficiency, as well as a decreased incidence of delivery room resuscitation and RDS.

Beyond its classical role in calcium homeostasis, vitamin D plays an important role in fetal lung maturation [18, 19]. Vitamin D receptors (VDRs) are expressed in fetal lung tissue, including alveolar epithelial cells, suggesting a direct regulatory role in lung development [20]. Experimental studies have shown that 1,25‐dihydroxyvitamin D promotes the differentiation of alveolar type II pneumocytes, which are responsible for surfactant synthesis [21]. Surfactant production is essential for reducing alveolar surface tension and preventing postnatal RDS [22].

Vitamin D has been shown to regulate the expression of surfactant‐associated proteins (SP‐A, SP‐B, and SP‐C) and to increase phospholipid synthesis in developing lungs [18, 21]. Animal models also show that vitamin D deficiency impairs alveolar maturation and reduces surfactant production, whereas supplementation restores normal lung structure and function [19].

Given that placental transfer of vitamin D occurs primarily during the third trimester of pregnancy, premature infants are particularly susceptible to deficiency [23]. Therefore, maternal vitamin D supplementation may contribute to improving neonatal respiratory outcomes by promoting structural and functional lung maturation [24].

The findings underscore the importance of vitamin D supplementation during preterm pregnancy, revealing significant differences in serum vitamin D levels and infant outcomes between mothers who were supplemented and those who were not. This finding aligns with existing literature that highlights the critical role of vitamin D in both maternal and neonatal health [25, 26]. Our results indicate that mothers supplemented with vitamin D had substantially higher serum vitamin D levels than those who did not. Likewise, preterm infant serum levels showed a similar trend. These findings are consistent with studies that confirm that vitamin D is transferred from the mother to the fetus during pregnancy, making maternal vitamin D status critical for preventing deficiency in the infant. Meta‐analysis findings revealed a significant increase in 25(OH)D levels in the umbilical cord blood by an average of 8.99 ng/mL (22.48 nmol/L) when mothers were supplemented with vitamin D [27].

Vitamin D deficiency is highly prevalent among pregnant women in Iran; a systematic review and meta‐analysis reported a pooled prevalence of 80.82% using a serum 25(OH)D cutoff of <30 ng/mL [28]. A systematic review of 17 studies involving pregnant and lactating women reported that the prevalence of vitamin D deficiency varies significantly by region, with rates as low as 4% in the United Kingdom and as high as 60% in India [29]. In the present study, the prevalence of severe vitamin D deficiency was considerably lower in supplemented mothers (21.4% vs. 46%) and their infants (36.4% vs. 62.2%), emphasizing the efficacy of vitamin D supplementation in addressing maternal and neonatal deficiencies. The substantial difference in normal vitamin D levels further reinforces the need for proactive vitamin D administration during pregnancy, particularly for those at risk of preterm delivery.

Infants born to mothers who took vitamin D showed improved serum levels and better clinical outcomes in several areas. For instance, the need for resuscitation in the delivery room was significantly greater among infants born to nonsupplemented mothers. Similarly, a study conducted in Ireland on preterm infants born at less than 32 weeks of gestation found that 25(OH)D levels below 12 ng/mL at the time of delivery were associated with increased oxygen demand, prolonged duration of mechanical ventilation with intermittent positive pressure in the delivery room, and a higher likelihood of requiring mechanical ventilation [30]. This finding highlights the potential risk factors associated with low vitamin D levels, as vitamin D supports various physiological processes that mitigate distress during delivery.

Although first‐ and fifth‐minute Apgar scores and birth weights did not differ significantly between the groups, this could indicate that while immediate clinical aspects of neonatal transition may not be affected by vitamin D status, more subtle and longer term effects may still exist. This notion is supported by our findings regarding adverse outcomes, where we noted significantly higher rates of respiratory problems and RDS in the nonsupplementation group. The detrimental effects of vitamin D deficiency on respiratory health are well‐documented [31]. A recent meta‐analysis showed that lower vitamin D levels or deficiencies within 24 h of birth were consistently linked to the development of RDS [32]. Another study found that infants with RDS had lower average serum vitamin D levels than those without respiratory difficulties, along with their mothers′ vitamin D levels being lower as well [33]. Additionally, research related to preterm births indicated a higher incidence of RDS among newborns with vitamin D insufficiency compared to those with adequate levels [31]. Infants born at a gestational age of less than 29 weeks and with blood 25(OH)D levels at or below 75 nmol/L experienced worse early outcomes, including increased oxygen requirements for RDS and extended mechanical ventilation during the first week of life, relative to those with sufficient vitamin D levels [34]. Recent studies highlight the importance of vitamin D in lung development, particularly in the synthesis of surfactant by alveolar Type II cells [35, 36]. Fetal vitamin D comes from the mother during the third trimester, with umbilical cord blood reflecting fetal levels. Vitamin D deficiency in mothers leads to impaired lung development in offspring, resulting in fewer alveoli, increased airway issues, and higher neonatal mortality. Children of vitamin D‐deficient mothers are also more likely to experience asthma and lung dysfunction. In animal studies, maternal vitamin D supplementation can improve lung outcomes, suggesting low vitamin D may delay lung maturation in preterm infants. These findings suggest that maternal vitamin D sufficiency could play a pivotal role in reducing such risks in preterm infants [37].

Despite our efforts to control for potential confounders, residual confounding cannot be excluded as a potential explanation for the observed associations.

The implications of our study are profound. Healthcare providers should consider implementing routine screening for vitamin D levels in women at risk of preterm labor, along with recommending supplementation where needed. It is particularly crucial given the high incidence of vitamin D deficiency observed within our study population and its associations with adverse maternal and neonatal outcomes.

While our study provides valuable insights, it is important to acknowledge certain limitations due to the single‐center design. As this is a cross‐sectional study, causal relationships cannot be established; the findings provide indications for further research, such as longitudinal studies to confirm causality. Supplement use was self‐reported, and details regarding timing during pregnancy and adherence were limited. This may introduce recall and misclassification bias. The minimum supplementation duration of 1 month may be insufficient to fully capture long‐term effects, as discussed in the literature, potentially leading to misclassification in the supplementation group. Limitations include unmeasured confounders like maternal BMI and sunlight exposure. Generalizability is limited by the single‐center design in Iran; findings may not apply to other regions.

Additionally, further research with larger sample sizes and longitudinal tracking of maternal and neonatal outcomes postdelivery is needed to better understand the long‐term effects of vitamin D supplementation.

### 5.1. Clinical Implications for Preterm Neonatal Care

Maternal vitamin D supplementation of at least 1000 IU/day for at least 1 month was associated with higher maternal and neonatal serum vitamin D levels and lower frequencies of respiratory complications as well as respiratory problems in preterm neonates. Therefore, maternal vitamin D supplementation may help decrease complications in preterm infants at birth.

## 6. Conclusion

In conclusion, this study, conducted in Iran (Asia), suggests an association between maternal vitamin D supplementation and higher serum vitamin D levels in both mothers and their preterm infants, potentially reducing the prevalence of severe deficiency and certain adverse neonatal outcomes in Asian populations with high deficiency rates. Given the adverse effects associated with lower vitamin D levels, these findings advocate for implementing vitamin D supplementation as a standard recommendation for pregnancy to optimize maternal and neonatal health outcomes, particularly in pregnancies at risk of preterm deliveries.

## Author Contributions

Conception and design, conceptualization, funding acquisition, methodology, project administration, and supervision: Hassan Boskabadi. Acquisition of data, data curation, investigation, software, resources, and visualization: Hassan Boskabadi, Maryam Zakerihamidi, and Fahimeh Afzaljavan. Analysis and interpretation of data, formal analysis, and validation. Hassan Boskabadi and Maryam Zakerihamidi. Drafting the manuscript and writing—original draft: Hassan Boskabadi, Maryam Zakerihamidi, Forough Rakhshanizadeh, and Fahimeh Afzaljavan. Critically revising the manuscript and writing—review and editing. Hassan Boskabadi, Maryam Zakerihamidi, and Forough Rakhshanizadeh. Final approval of the version to be published: Hassan Boskabadi, Maryam Zakerihamidi, and Forough Rakhshanizadeh. Agreement to be accountable for all aspects of the work in ensuring that questions related to the accuracy or integrity of any part of the work are appropriately investigated and resolved: Hassan Boskabadi, Maryam Zakerihamidi, Forough Rakhshanizadeh, and Fahimeh Afzaljavan.

## Funding

No funding was received for this manuscript.

## Disclosure

The present study is the result of the Mashhad University of Medical Sciences–approved project.

## Ethics Statement

The study protocol was reviewed and approved in accordance with the Declaration of Helsinki by the Ethics Committee of Mashhad University of Medical Sciences, Mashhad, Iran (Ethical Approval Code: IR.MUMS.MEDICAL.REC.1399.204).

## Consent

All participants signed the consent form after receiving complete information regarding the study procedure and goals. Confidentiality and anonymity were strictly maintained, and participants were assured that their involvement was entirely voluntary, with the right to withdraw from the study at any time without any impact on their standard of care or medical treatment.

## Conflicts of Interest

The authors declare no conflicts of interest.

## Supporting information


**Supporting Information** Additional supporting information can be found online in the Supporting Information section. The completed STROBE checklist for cross‐sectional studies is provided as supporting information (STROBE_checklist_cross‐sectional.docx).

## Data Availability

Data will be made available upon request.
